# Factors influencing bone mineral density in hyperparathyroidism phenotypes: a prospective study

**DOI:** 10.3389/fendo.2025.1562340

**Published:** 2025-05-28

**Authors:** Margot Giocondo, Belén Ponte, Sophie de Seigneux, Samira M. Sadowski, Benoît Bédat, Frédéric Triponez, Marco Stefano Demarchi

**Affiliations:** ^1^ Department of Thoracic and Endocrine Surgery and Faculty of Medicine, University Hospitals of Geneva, Geneva, Switzerland; ^2^ Nephrology and Hypertension Division, Department of Medicine, University Hospitals of Geneva, Geneva, Switzerland; ^3^ Neuroendocrine Cancer Therapy Section, Surgical Oncology Program, Center for Cancer Research, National Cancer Institute, National Institutes of Health, Bethesda, MD, United States

**Keywords:** primary hyperparathyroidism, bone mineral density, parathyroidectomy, dual-energy x-ray absorptiometry, normocalcemic hyperparathyroidism

## Abstract

**Background:**

Primary hyperparathyroidism (PHPT) is a condition recognized to include distinct biochemical phenotypes: hypercalcemic, normocalcemic, and normohormonal PHPT. This condition is characterized by inappropriately elevated parathyroid hormone (PTH) levels relative to serum calcium levels, leading to high bone turnover and decreased bone mineral density (BMD). Although BMD has been shown to improve following curative parathyroidectomy (PTX), there is limited data on the relationship between BMD changes after PTX and according to the clinical phenotypes of PHPT. This prospective study aims to identify the best candidates for surgery among these three phenotypes of PHPT by examining symptoms and comorbidities, biochemical results, and both pre- and post-operative DXA scans.

**Methods:**

This is a prospective single-center study including 104 consecutive patients who underwent PTX for PHPT. Sociodemographic profiles and biochemical results were collected at both pre- and at 6 months post-surgery. All patients underwent dual-energy x-ray absorptiometry (DXA) scans at 1 and 6 months before surgery, and again between 12 and 18 months after surgery, to assess and compare changes in bone mineral density (BMD). Patients were categorized into three subgroups (normohormonal, hyperparathyroid normocalcemic and hyperparathyroid hypercalcemic) to assess differences in BMD improvement across these groups.

**Results:**

According to predefined thresholds, 40.0% of patients experienced a significant BMD gain at the spine, 35.1% at the femur, 23.5% at the left hip, and 8.6% at the radius. Additionally, a greater postoperative drop in PTH was associated with larger BMD gains at the spine (SD 0.07; mean 0.03) and left femur bone (SD 0.06; mean 0.02). There was no significant change in T-score at the one-third radius. Higher preoperative urinary calcium level was associated with greater BMD and T-score improvement at the left hip. All PHPT phenotypes demonstrated similar postoperative BMD gains. No differences in BMD improvement were observed between males and females, nor was there any correlation between BMD gains and age.

**Conclusions:**

Our findings suggest that BMD improves in all PHPT patients undergoing curative PTX, irrespective of clinical phenotype, age or sex. While patients with more severe biochemical profiles exhibited greater increases in BMD and T-scores, milder forms showed favorable trends that require confirmation in larger studies.

## Introduction

Primary hyperparathyroidism (PHPT) is a common endocrine disorder characterized by parathyroid hormone (PTH) levels inappropriately elevated relative to serum calcium levels, typically due to overactivity in one or more parathyroid glands. Since the 1970s, advances in biochemical screening have expanded the understanding of PHPT from its classical presentation—associated with significant hypercalcemia, bone pain, kidney stones, and neuropsychiatric symptoms—to a spectrum of clinical phenotypes (1, 2). These include “asymptomatic” PHPT (3), where patients present with slightly elevated or upper-normal serum calcium levels but with no obvious clinical signs of PHPT, and normocalcemic PHPT (4–6), where patients have persistently elevated PTH levels with normal adjusted and ionized calcium in the absence of secondary causes.

One consequence of elevated PTH secretion in PHPT is increased bone turnover (7), which leads to decreased bone mineral density (BMD), particularly at cortical sites (8). This reduction in BMD can result in osteopenia or osteoporosis in the long run, thereby increasing the risk of fractures. Numerous studies have shown that patients with PHPT experience an increase in BMD following successful parathyroidectomy (PTX) (9–13). Several factors are thought to influence the extent of BMD improvement. Randomized clinical trials have documented BMD improvements in patients with mild, asymptomatic PHPT (14), yet a meta-analysis indicated that surgery offers similar benefits compared to surveillance (15, 16), albeit with the need for longer follow-up periods. Moreover, recent research has highlighted that even normocalcemic PHPT patients experience significant BMD gains post-PTX (17, 18). Additionally, differences in patients’ sociodemographic profiles, such as age, and biochemical characteristics, like hypercalciuria or lower baseline PTH, are predictors of better BMD improvement (19).

Although distinct phenotypes of PHPT have emerged, PTX remains the only curative treatment, with growing evidence supporting its efficacy in improving BMD in PHPT patients. PTX is recommended for all symptomatic and asymptomatic cases with serum calcium ≥1 mg/dl above the upper limit of normal, those with osteoporosis (T-score ≤−2.5) or vertebral fractures, those with eGFR <60 ml/min, severe hypercalciuria (>400 mg/day), increased risk of stones, evidence of nephrolithiasis, or nephrocalcinosis, and those under 50 years of age (3, 17). However, with the evolving definition of the disease and limited data on the different phenotypes, the question of which patients should undergo surgery remains. This study focuses on the predictors of increased postoperative BMD across different PHPT phenotypes.

## Materials, methods and definition

### Study design and population

We performed a prospective observational study including patients diagnosed with primary hyperparathyroidism (PHPT), who underwent parathyroidectomy at Geneva University Hospitals in Geneva, Switzerland, between November 2016 and May 2020.

### Inclusion and exclusion criteria

Inclusion criteria: Patients aged 18 years or older diagnosed with primary hyperparathyroidism who provided written informed consent for participation in the study.

Exclusion criteria: Patients were excluded if they met any of the following conditions: (1) diagnosis of secondary or tertiary hyperparathyroidism, (2) absence of preoperative bone mineral density (BMD) measurements, or (3) failure to meet the inclusion criteria.

This study was conducted in accordance with the guidelines outlined in the Declaration of Helsinki. All procedures involving research participants were approved by the Ethics Committee, and informed consent was obtained from all subjects involved in the study.

### Collected variables

Demographic and clinical data were collected during the initial preoperative visit: age, sex, height, weight, BMI and clinical symptoms like osteopenia and osteoporosis.

For the laboratory measures, serum and urinary calcium, parathyroid hormone (PTH), and phosphate levels were evaluated at multiple time points throughout the study. Preoperative measurements were taken during the initial visit (V1). Postoperative assessments were conducted on day 1 (V2) and day 10 (V3) following parathyroidectomy (PTX), with a final follow-up at 6 months post-surgery (V4). The analytical methods employed were specific to each biomarker. PTH was quantified using the Cobas 8000 ECLIA (Electrochemiluminescence immunoassay) method, with normal values ranging from 1.1 to 6.8 pmol/L. Both serum calcium and phosphate were analyzed using UV-visible absorption spectrometry. The reference range for serum calcium was established between 2.20 and 2.52 mmol/L, while phosphate levels were considered normal between 0.80 and 1.45 mmol/L.

Bone mineral density (BMD) was assessed using dual-energy x-ray absorptiometry (DXA) in g/cm^2^ at the same facility and with the same machine for all patients. Scans were performed by trained technicians. Measurements were conducted between 1 and 6 months prior to surgery and again between 12 and 18 months post-surgery. Bone density and T-scores were measured at spine, left femur, left hip and radius.

Regarding the preoperative imaging protocol, a comprehensive evaluation was performed using two complementary techniques: high-resolution neck ultrasound and technetium-99m sestamibi scintigraphy. This dual-modality approach was employed to accurately localize parathyroid adenomas and guide surgical planning.

Finally, we collected intraoperative data: identified parathyroid glands number, removed glands number and weight.

### Definitions

The three biochemical profiles were defined based on lab values at the first visit (V1) as follows: the normohormonal (NH) group had normal PTH with elevated serum calcium, the hyperparathyroid normocalcemic (HC) group had elevated PTH with normal serum calcium, and the hyperparathyroid hypercalcemic (HH) group exhibited concomitantly elevated serum calcium and PTH. This classification was adopted based on previous studies (25) highlighting distinct biochemical patterns and potentially different pathophysiological mechanisms among these subtypes.

The changes in BMD and T-scores were determined by subtracting preoperative values from postoperative values. Declining in bone density is considered <0%, moderate improvement is considered between 0.1 and 5%, and significant improvement with more than 5% (20).

### Statistical analysis

The distribution of demographic, preoperative characteristics, and symptom variables was analyzed to identify patterns and potential outliers. Histograms, box plots, and normality tests (e.g., Shapiro-Wilk test) were used to assess the shape of distributions. IBM SPSS software was used to perform all statistical analyses.

Comparative analyses were conducted to explore differences in preoperative characteristics and cross demographic subgroups, utilizing t-tests or ANOVA for continuous variables and chi-square tests for categorical variables. Pearson and Spearman correlation coefficients were calculated to assess the relationships between demographic variables, preoperative characteristics, symptoms, and the changes in BMD and T-scores from pre- to postoperative measurements.

## Results

We initially included 123 patients, but only 111 completed the study and follow-up, from which 104 had BMD measures at the 2 time points. Therefore, for this study we analyzed 104 PHPT patients from whom 84 were females (80.7%) and 20 males (19.2%). Their baseline characteristics are reported in **Table 1** and **Table 2**. They were divided into three categories according to serum calcium and PTH levels (see **Table 3**). A total of 5 (4.8%) were classified as normohormonal hypercalcemic (NH), 32 (30.8%) as hyperparathyroid normocalcemic (HC), and 67 (64.4%) as hyperparathyroid hypercalcemic (HH). According to Charlston Comorbidity Index, 19.2% had no comorbidities and 85.6% had one and more. Out of 104 patients, 44% had osteopenia and 37.5% had osteoporosis. By subgroup, osteopenia was present in 40% of NH, 40.6% of HC, and 46.2% of HH patients, while osteoporosis was found in 40%, 46.9%, and 32.8% respectively (see **Table 2**).

**Table 1:** clinical baseline characteristics of the patients included in the study.

Variable	N	Value (% or SD)
Female Gender	84	80.7%
Age (years)	104	62.6 ± 14.8
Caucasian Ethnicity	96	92.3%
Weight (kilograms)	104	71.0 ± 14.3
Height (centimeters)	104	165.3 ± 8.2
BMI (kg/m2)	104	24.6
Smoker	23	22.1%
Hypertension	36	34.6%
Charlston Comorbidity Index		
0	20	19.2%
1	18	17.3%
2	32	30.8%
3	27	26%
4	12	11.5%
PHPT subgroups	N	Value
Normohormonal hypercalcemic (NH)	5	4.8%
Hyperparathyroid normocalcemic (HC)	32	30.8%
Hyperparathyroid hypercalcemic (HH)	67	64.4%

All continuous variables are presented with appropriate measurement units. SD, standard deviation; BMI, Body Mass Index; PHPT, Primary Hyperparathyroidism; IQR, InterQuartile range (25-75%).

**Table 2:** Osteopenia and osteoporosis percentages in the three groups.

		PHPT phenotypes					
		Normohormonal hypercalcemic		Hyperparathyroid hypercalcemic		Hyperparathyroid normocalcemic	
		n	%	n	%	n	%
**Bone status**	**Osteopenia**	2	40	31	46.2	13	40.6
	**Osteoporosis**	2	40	22	32.8	15	46.9

**Table 3:** Pre-operative calcium and PTH concentrations in the 3 subgroups (V1).

		PHPT phenotypes					
		Normohormonal hypercalcemic		Hyperparathyroid hypercalcemic		Hyperparathyroid normocalcemic	
		n	Median (mmol/L)	n	Median (mmol/L)	n	Median (mmol/L)
**Serum levels**	**Calcium (total)**	5	2.68	67	2.75	32	2.55
	**Calcium (corrected)**	5	2.58	67	2.64	32	2.45
	**PTH**	5	5.71	67	13.10	32	9.88

The median time interval between preoperative and postoperative bone density measurements was 1.67 years [IQR 1.23 - 2.10], and the median time interval between the last bone density measurement and surgery was 1.06 years [IQR 0.95 - 1.4].

### Imaging

Considering imaging and surgical findings of patients, assessment of glands by cervical US showed that one gland was more prevalent in HH patients (*p* = 0.026), with median gland weight also significantly higher in this group (median = 0.82 [IQR 0.33 – 1.43] (*p* < 0.001), that is 4-fold higher than the other groups as well. This is also linked with histology findings that showed adenoma in 75% of the cases, double adenoma in 7.8%, and hyperplasia in 22.1% of the patients (**Supplementary Table 1**).

### Surgical management

We identified discordant preoperative imaging results in 36.5% of patients, between high-resolution neck ultrasound and technetium-99m sestamibi scintigraphy. Bilateral surgical exploration was performed in 35.6% of patients. The majority of patients (80.8%) had one gland removed, while 17.3% had two and 6.7% had three glands removed (**Supplementary Table 1**).

### Biological modifications after PTX

Analysis of total calcium and corrected calcium at V1 showed a significant difference in pre-operative calcium between the groups, with highest values, by definition, in the HH patients and lowest values in NH patients (p < 0.001). As expected, there were significant differences in preoperative PTH between NH, HC or HH groups. Preoperative (V1) total calcium was significantly different (p < 0.001) between HC and HH groups, with a median total calcium of 2.55 and 2.75 mmol/l, respectively (see **Table 3**).

### BMD evolution after PTX

Post-operative BMD was performed at a median of 1.05 years after surgery. Pre- and post-operative DXA scans with resulting bone mineral density parameters and calculated T-scores of the patients summarized in **Table 4**. When comparing preoperative and post-operative BMD, PTX was associated with a significant increase in BMD at the spine (standard deviation (SD) 0.07; mean 0.03), left femur bone (SD 0.06; mean 0.02), and at the left hip bone (SD 0.06; mean 0.01), whereas no significant difference was found at the one-third radius (**Table 5**).

**Table 4:** Preoperative and postoperative bone density parameters of the patients included in the study.

Site	Measurement	Preoperative	Postoperative	Difference	Effect Size (ES)	P-value
Spine	Bone Density	0,906 ± 0.165	0.934 ± 0.153	0.027 ± 0.072	0,165	0,001
	T-score	-1,406 ± 1.355	-1.095 ± 1.34	0.262 ± 0.562	0,193	0,001
Left Hip	Bone Density	0,824 ± 0.124	0.836 ± 0.121	0.013 ± 0.058	0,105	0,03
	T-score	-1,043 ± 0.932	-0.96 ± 0.916	0.081 ± 0.467	0,086	0,091
Left Femur	Bone Density	0,694 ± 0.117	0.717 ± 0.117	0.021 ± 0.057	0,175	0,001
	T-score	-1,545 ± 0.964	-1.363 ± 0.915	0.166 ± 0.352	0,173	0,001
Radius	Bone Density	0,634 ± 0.122	0.617 ± 0.123	-0.008 ± 0.045	-0,063	0,194
	T-score	-1,528 ± 1.73	-1.627 ± 1.736	-0.074 ± 0.923	-0,042	0,543

Bone density is expressed in g/cm².

**Table 5:** Paired differences in BMD (g/cm²) between preoperative and postoperative measurements at four skeletal sites.

Site	Mean Change (g/cm²)	Standard Deviation (SD)	95% CI (Lower–Upper)	P-value (Two-Sided)
Spine	0.0267	0.0724	0.0123 – 0.0411	< 0.001
Left hip	0.0130	0.0582	0.0013 – 0.0246	0.030
Left femur	0.0207	0.0571	0.0092 – 0.0323	0.001
Radius	–0.0078	0.0451	–0.0197 – 0.0041	0.194

Positive values indicate an increase in BMD after PTX. p-values refer to paired t-tests. CI, confidence interval.

**Figure 1:**
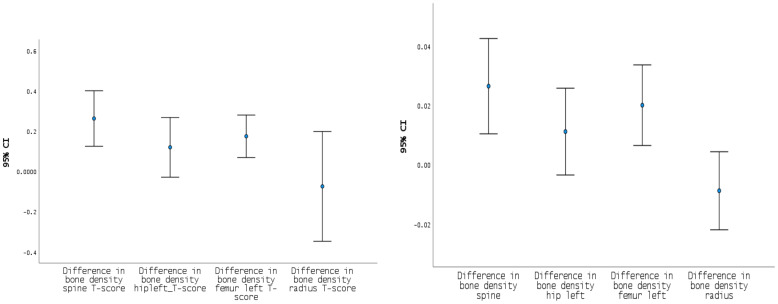
IC 95% difference in T-score and bone density for spine, left hip, left femur and radius. Mean changes in T-score (left) and BMD (right) at the spine, left hip, femur, and radius one year after parathyroidectomy. The most significant improvements were observed at the spine, while no significant change was detected at the radius. At the left hip, changes were more modest, with confidence intervals crossing zero, indicating weaker statistical significance.

**Figure 1** and its **Supplementary Table 2** illustrate the 95% confidence intervals for changes in bone mineral density (BMD) and T-scores at the spine, left femur, left hip, and one-third radius. The spine showed the most significant and consistent gains in both BMD and T-score. Improvements at the left femur were also notable. At the left hip, changes were more modest, with confidence intervals crossing zero, indicating no statistical significance. No significant change was observed at the one-third radius, which also demonstrated the greatest variability—some patients experienced substantial gains, while others showed no improvement or even loss.

**Figure 2:**
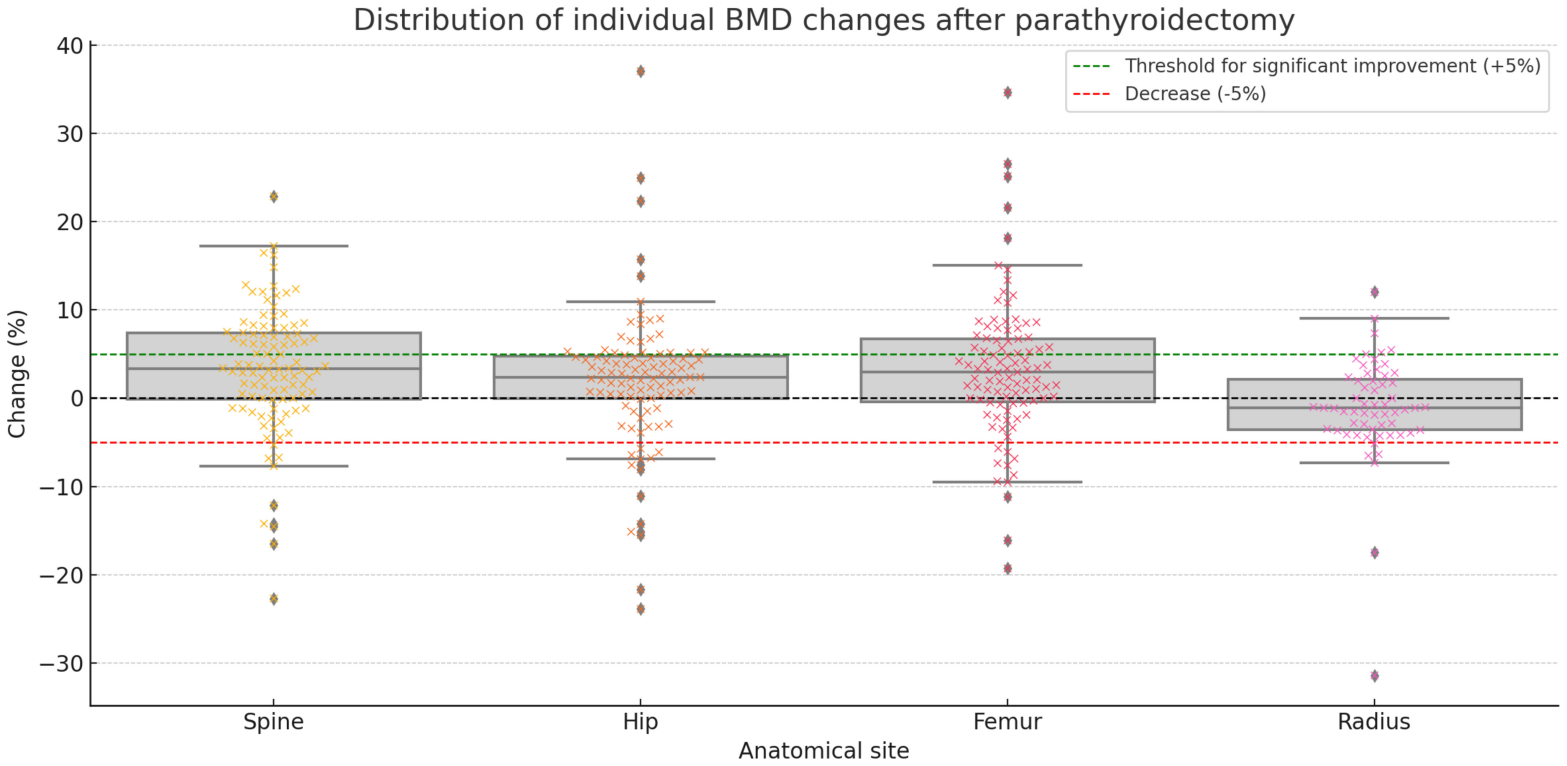
Distribution of individual changes in bone mineral density (BMD) for all patients at the spine, left hip, left femur, and radius after parathyroidectomy. Each dot represents an individual patient. Box plots show the distribution of percent changes. The green dashed line indicates the +5% threshold for significant improvement, while the red dashed line marks a 5% decrease. The black dashed line represents no change. For example, in the spine, 27.0% of patients showed a decrease, 33.0% had a moderate increase (0.1–5%), and 40.0% had a significant increase (>5%).

In addition to statistical comparisons, individual-level changes in BMD were evaluated according to the predefined threshold of a >5% increase representing a significant improvement. As illustrated in **Figure 2**, which shows the distribution of percent BMD change at each anatomical site, a significant improvement (>5%) in BMD was observed in 40.0% of patients at the spine, 35.1% at the left femur, 23.5% at the left hip, and 8.6% at the radius. Moderate increases (0.1–5%) and decreases were also observed across all sites, highlighting the heterogeneity in individual responses to surgery.

**Table 6** presents the Spearman’s rho correlations between preoperative PTH levels (V1) and postoperative changes in BMD and T-scores at various anatomical sites. Our analysis showed modest but statistically significant associations between baseline PTH and BMD improvements, especially at the spine (ρ = 0.240, *p* = 0.017) and left femur (ρ = 0.335, *p* = 0.001), as well as their corresponding T-scores (spine: ρ = 0.241, *p* = 0.017; left femur: ρ = 0.220, *p* = 0.032). These associations suggest that patients with higher baseline PTH may exhibit greater postoperative gains in BMD at these sites. Higher preoperative urinary calcium levels were significantly associated with greater improvements in both BMD and T-score at the left hip, as shown in **Table 7**.

**Table 6:** Spearman’s rho correlation between PTH levels at V1 to V4 and BMD.

		Difference in bone density					Difference in T-score			
			Spine	Hip left	Femur left	Radius	Spine	Hip left	Femur left	Radius
**Serum PTH for each visit**	**V1 PTH**	Correlation Coefficient	.240^*^	0,182	.335^**^	0,196	.241^*^	0,153	.220^*^	0,106
		p-value	0.017	0,075	0.001	0,140	0.017	0,136	0.032	0,427
		N	98	97	95	58	97	97	95	58
	**V2 PTH**	Correlation Coefficient	-.252^*^	-0,095	0,042	-0,162	-0,189	-0,158	0,168	-0,121
		p-value	0.012	0,353	0,684	0,224	0,063	0,123	0,102	0,367
		N	99	97	96	58	98	97	96	58
	**V3 PTH**	Correlation Coefficient	0,022	0,046	0,117	-0,017	0,004	0,050	0,133	0,001
		p-value	0,824	0,651	0,254	0,901	0,970	0,628	0,195	0,994
		N	100	98	97	58	99	98	97	58
	**V4 PTH**	Correlation Coefficient	-0,071	0,004	0,073	0,099	-0,116	0,012	0,043	0,010
		p-value	0,489	0,966	0,485	0,466	0,263	0,906	0,681	0,945
		N	96	95	93	56	95	95	93	56

**Correlation is significant at the 0.01 level (2-tailed). *Correlation is significant at the 0.05 level (2-tailed).

**Table 7:** Spearman’s rank correlation coefficients between urinary calcium levels at each visit and change in BMD at various skeletal sites.

Site	Visit	Spearman's rho	p-value	95% CI (Lower, Upper)
Spine	V1	0.124	0.238	–0.084, 0.322
	V2	0.021	0.849	–0.194, 0.235
	V3	–0.075	0.473	–0.273, 0.130
	V4	–0.128	0.213	–0.321, 0.075
Left hip	V1	**0.269**	**0.009**	**0.065, 0.452**
	V2	0.134	0.232	–0.087, 0.341
	V3	0.006	0.952	–0.197, 0.209
	V4	0.177	0.088	–0.028, 0.368
Left femur	V1	0.157	0.138	–0.052, 0.352
	V2	–0.036	0.749	–0.253, 0.184
	V3	**0.213**	**0.042**	**0.006, 0.402**
	V4	0.012	0.909	–0.191, 0.214
Radius	V1	0.263	0.055	–0.008, 0.497
	V2	0.282	0.052	–0.005, 0.526
	V3	0.047	0.731	–0.219, 0.307
	V4	0.101	0.455	–0.163, 0.35

Statistically significant associations were observed at the left hip at V1 and the left femur at V3. No significant correlation was found at the spine or radius. Values in bold indicate p < 0.05.

Bold values indicate statistically significant results (p < 0.05).

When comparing variation in bone density by gender, no significant difference was found between males and females. We also found no significant differences in bone density levels between the three hormonal subgroups, which aligns with current literature. **Figure 2** and **Table 8** demonstrate that patients across all three phenotypes—hypercalcemic, normocalcemic, and normohormonal—experienced BMD improvements, with comparable proportions achieving moderate or significant gains.

**Table 8:** Variation in bone density, overall, and by gender.

	Variation in bone density		Declining bone density (<0%)		Moderate improvement in bone density (0.1%-5%)		Significant improvement in bone density (>5%)		p-value
			n	%	n	%	n	%	
**BMD accross anatomical sites**	**Spine**	**All**	27	27.0%	33	33.0%	40	40.0%	0.411
		**Male**	3	16.7%	8	44.4%	7	38.9%	
		**Female**	24	29.3%	25	30.5%	33	40.2%	
	**Hip left**	**All**	26	26.5%	49	50.0%	23	23.5%	0.475
		**Male**	4	21.1%	12	63.2%	3	15.8%	
		**Female**	22	27.8%	37	46.8%	20	25.3%	
	**Femur left**	**All**	28	28.9%	35	36.1%	34	35.1%	0.417
		**Male**	4	22.2%	9	50.0%	5	27.8%	
		**Female**	5	41.7%	6	50.0%	1	8.3%	
	**Radius**	**All**	37	63.8%	16	27.6%	5	8.6%	0.140
		**Male**	24	30.4%	26	32.9%	29	36.7%	
		**Female**	32	69.6%	10	21.7%	4	8.7%	
**T-scores accross anatomical sites**	**Spine T-score**	**All**	79	79.8%	2	2.0%	18	18.2%	0.462
		**Male**	13	72.2%	0	0.0%	5	27.8%	
		**Female**	66	81.5%	2	2.5%	13	16.0%	
	**Hip left T-score**	**All**	68	69.4%	1	1.0%	29	29.6%	0.268
		**Male**	10	52.6%	0	0.0%	9	47.4%	
		**Female**	58	73.4%	1	1.3%	20	25.3%	
	**Femur left T-score**	**All**	79	82.3%	1	1.0%	16	16.7%	0.999
		**Male**	15	83.3%	0	0.0%	3	16.7%	
		**Female**	64	82.1%	1	1.3%	13	16.7%	
	**Radius T-score**	**All**	27	47.4%	4	7.0%	26	45.6%	**0.024**
		**Male**	10	83.3%	0	0.0%	2	16.7%	
		**Female**	17	37.8%	4	8.9%	24	53.3%	

Patients were categorized based on the percentage change between pre- and post-operative measurements into three groups: decrease (<0%), moderate increase (0.1–5%), and significant increase (>5%). The data show BMD improvements across all measured sites, with a substantial proportion of patients achieving moderate or significant gains, regardless of biochemical phenotype. These findings support the observation that parathyroidectomy benefits all patients with PHPT, irrespective of their initial biochemical profile.

To further evaluate whether BMD changes differed across the three biochemical phenotypes of PHPT (hypercalcemic, normocalcemic, and normohormonal), we conducted ANOVA tests on preoperative, postoperative, and delta BMD values at all anatomical sites. As shown in **Table 9**, no statistically significant differences were found between the groups, either at baseline, after surgery, or in terms of BMD change. These findings confirm that the magnitude of bone mineral density improvement following parathyroidectomy is comparable across all three clinical phenotypes.

**Table 9:** Comparison of the 3 groups using ANOVA tests.

		Normohormonal hypercalcemic				Hyperparathyroid normocalcemic				Hyperparathyroid hypercalcemic				
		n	Mean	sd	IC 95%	n	Mean	sd	IC 95%	n	Mean	sd	IC 95%	p-value
**Preoperative BMD**	**Spine**	5	0.93	0.22	0.74 - 1.12	32	0.87	0.13	0.83 - 0.92	62	0.92	0.18	0.87 - 0.96	0.471
	**Hip left**	5	0.89	0.18	0.74 - 1.05	31	0.81	0.12	0.77 - 0.86	63	0.82	0.12	0.79 - 0.85	0.402
	**Femur left**	5	0.77	0.16	0.63 - 0.91	31	0.70	0.10	0.66 - 0.73	58	0.68	0.12	0.65 - 0.72	0.288
	**Radius**	2	0.76	0.05	0.70 - 0.83	16	0.60	0.13	0.54 - 0.67	43	0.64	0.12	0.61 - 0.68	0.185
**Postoperative BMD**	**Spine**	5	0.94	0.21	0.76 - 1.12	31	0.90	0.13	0.86 - 0.95	63	0.95	0.16	0.91 - 0.99	0.484
	**Hip left**	5	0.91	0.16	0.77 - 1.05	31	0.83	0.12	0.79-0.87	61	0.84	0.12	0.81 - 0.87	0.359
	**Femur left**	5	0.77	0.15	0.64 - 0.90	31	0.71	0.11	0.67 -0.75	63	0.71	0.12	0.68 - 0.74	0.513
	**Radius**	4	0.75	0.04	0.71 - 0.79	29	0.62	0.14	0.57 - 0.67	51	0.61	0.12	0.58 - 0.64	0.105
**Difference in BMD**	**Spine**	5	0.01	0.06	-0.04 - 0.062	31	0.03	0.06	0.01 - 0.05	59	0.03	0.08	0.01 - 0.05	0.789
	**Hip left**	5	0.02	0.04	-0.02 - 0.06	31	0.01	0.06	-0.01 - 0.03	59	0.02	0.05	0.01 - 0.03	0.992
	**Femur left**	5	0.00	0.07	-0.06-0.06	31	0.01	0.07	-0.01 - 0.03	56	0.03	0.05	0.02 - 0.04	0.500
	**Radius**	2	0.00	0.01	-0.01-0.01	16	0.00	0.07	-0.03 - 0.03	38	-0.01	0.03	-0.02 - 0.0	0.922

None of the comparisons were statistically significant (all p > 0.05), supporting that BMD improvements following PTX occur consistently across clinical subtypes.

This consistent pattern supports our conclusion that parathyroidectomy is beneficial for PHPT patients regardless of their biochemical profile.

## Discussion

**Figure 3:**
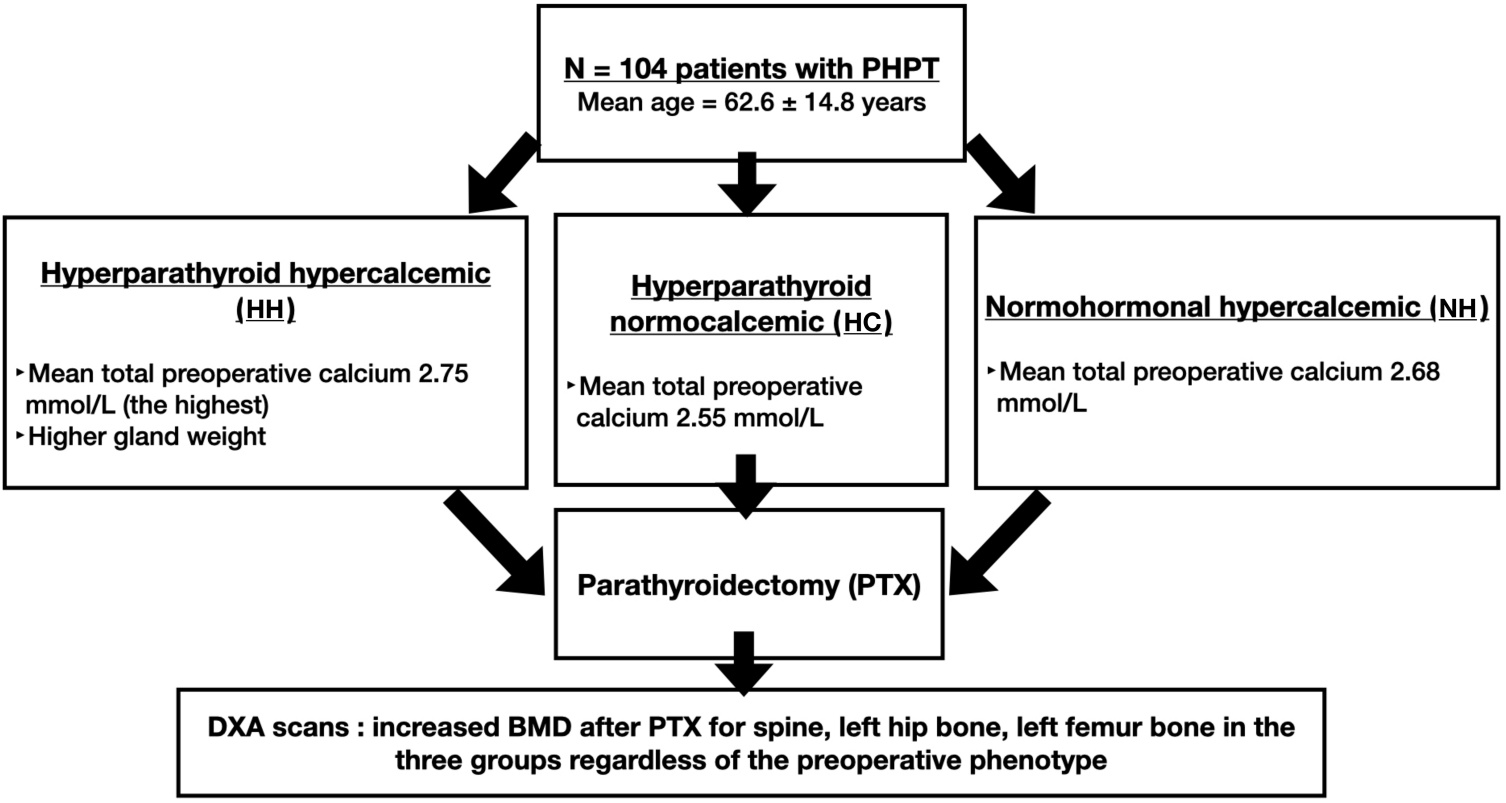
Findings’ summary of the comparison of the 3 groups. Patients from all three phenotypes—hypercalcemic, normocalcemic, and normohormonal—experienced postoperative BMD gains. These improvements were consistent across skeletal sites, with no significant differences between groups, supporting the role of PTX in all PHPT variants.

This prospective study of 104 patients with primary hyperparathyroidism (PHPT) who underwent curative parathyroidectomy (PTX) at Geneva University Hospitals confirms and extends prior evidence that BMD improves after surgery. Notably, we demonstrate that this benefit applies consistently across the three major biochemical phenotypes of PHPT—hypercalcemic, normocalcemic, and normohormonal. A visual summary of our conclusions can be found on **Figure 3**.

When comparing pre-operative and post-operative parathyroid hormone (PTH) levels, the greatest drop in PTH was linked to a greater rise in BMD and T-score both in the spine and left femur, as well as corresponding gains in T-scores (**Table 6**). As expected, no meaningful correlations were observed at later time points (V3 and V4), when both PTH and BMD had largely normalized. While this study focused on baseline PTH, future research could investigate the predictive value of the change in PTH (ΔPTH) as a dynamic marker of skeletal recovery following parathyroidectomy.

We acknowledge, however, that the strength of these associations varied across anatomical sites. While the correlations at the spine and left femur were statistically significant, the association at the left hip was not statistically significant, but demonstrated a trend suggestive of clinical relevance, and no correlation was observed at the one-third radius. The observed BMD improvement at these sites is consistent with observational and randomized studies (22, 23) and also reported in a recent study by Osorio-Silla et al. (11). In fact, differences between pre- and post-operative BMD showed the greatest variability at the radius; some patients showed an improvement in BMD whereas other patients showed a decline in BMD at this site. These findings are in line with the literature, with different studies reporting contrasting results; some studies report an increase (24), others no change (22) and others still report a decrease in BMD at the one-third radius (5, 13). This may highlight one of the limitations of our study: the follow-up period. With a median time interval between surgery and the last bone density measurement of 1.06 years [IQR 0.95 -1.4] this follow-up time is relatively short and may explain why we do not capture an improvement in BMD at this site. Longer-term data are likely needed to observe improvement or stabilization at the one-third radius after parathyroidectomy. However, extended follow-up remains logistically challenging in patients who are successfully cured of a benign disease.

An additional limitation with our study when assessing findings is the limited size of the normohormonal hypercalcemic group (n = 5), where we cannot exclude a type II error for differences observed in this group compared to the other two groups. We acknowledge that a subset of patients with hypercalcemic PHPT may present with inappropriately normal PTH levels, especially in mild cases. However, we chose to classify patients into three distinct phenotype since previous studies (25) have highlighted differences in biochemical profiles and potential underlying mechanisms among these groups. Even though the normohormonal group is small, we believe that presenting these patients separately provides transparency in our analysis and allows future studies to build on our findings.

While patients with more severe biochemical profiles exhibited the largest increases in BMD and T-scores, even asymptomatic individuals—particularly those in the normohormonal group—showed favorable trends following PTX. However, as previously noted, the small sample size in this subgroup (n = 5) limited statistical power, and most of the 95% confidence intervals for both the normohormonal and normocalcemic groups crossed zero (**Table 9**), precluding firm conclusions. This suggests that although the direction of change is encouraging, the magnitude of effect may not be clinically or statistically meaningful in all cases.

Higher preoperative urinary calcium was associated with a greater BMD and T-score gain in left hip (**Table 7**). This finding is in line with Lee et al. (13) who reported that the degree of elevation of preoperative calcium is associated with post-PTX change in BMD. Similarly, Benzon et al. (20) also found that patients with greater serum PTH and urinary calcium preoperatively benefited greater gains on BMD and T-score. As expected, high serum PTH and high calciuria are indicative of the more symptomatic form of PHPT.

In line with previous studies by Lee et al. (13)and Koumakis et al. (5, 21), our study also suggests that multiple nodules are more common in HC patients compared to the other two groups. Koumakis et al. (5) compared HC and HH patients and found that the former are more likely to have multinodular adenomas. In addition to gland number, analysis of gland weight in our study highlighted that whilst HH patients had fewer diseased glands (only one in nearly 90% of cases), gland weight was increased nearly fourfold compared to the other two groups. This finding suggests a positive correlation between PTH levels and gland size, with larger (heavier) glands secreting more PTH, as typically observed in more severe forms of the disease.

Taken together, our findings confirm that BMD improves significantly in PHPT patients undergoing PTX across biochemical phenotypes, age groups, and sexes. The absence of significant differences in BMD gains between subgroups (**Table 8**) supports the potential for broad surgical benefit. While our results must be interpreted in light of subgroup limitations, they support a more inclusive consideration of surgery for PHPT patients, including those with mild or atypical biochemical presentations. This prospective study provides compelling evidence that PTX offers skeletal benefits across the clinical spectrum of PHPT, reinforcing the need to revisit current surgical indications.

## Conclusion

This prospective study provides evidence that parathyroidectomy enhances bone mineral density in patients with primary hyperparathyroidism (PHPT), including those with normocalcemic and normohormonal biochemical profiles. Nearly 40% of patients achieved a BMD gain exceeding 5%, a threshold considered clinically meaningful in DXA monitoring. While the most substantial gains were observed in patients with more severe biochemical profiles, even those with milder or asymptomatic forms of PHPT demonstrated favorable trends.

However, the magnitude of BMD improvement in normohormonal and normocalcemic patients was modest, and the associated confidence intervals often included zero. This highlights the need for cautious interpretation of subgroup results and underscores the importance of confirming these findings in larger cohorts.

Overall, our findings support extending surgical consideration to a broader population of PHPT patients. Future studies should aim to clarify the long-term skeletal outcomes in normocalcemic and normohormonal subtypes, and to further refine surgical indications across the full spectrum of PHPT.

## Data Availability

The raw data supporting the conclusions of this article will be made available by the authors, without undue reservation.

## Supplementary material

Supplementary Table 1 Imaging and surgical characteristics of the patients included in the study.

Supplementary Table 2 IC 95% difference in T-score and bone density for spine, left hip, left femur and radius. Cohen's d uses the sample standard deviation of the mean difference adjusted by the correlation between measures. Hedges' correction uses the sample standard deviation of the mean difference adjusted by the correlation between measures, plus a correction factor.
